# Understanding Responsible Antimicrobial Practices and Antimicrobial Resistance: A Cross‐Sectional Study of Farmers in Rangpur, Bangladesh

**DOI:** 10.1002/mbo3.70308

**Published:** 2026-05-06

**Authors:** Anju Man Ara, Farjana Boby, Mohsin Taluckder, Md. Shahiduzzaman

**Affiliations:** ^1^ Department of Food and Nutritional Science International Institute of Applied Science and Technology Rangpur Bangladesh; ^2^ The Wellington Academy; Commonwealth Shared Scholar; ^3^ One Health and Climate Smart Research Center International Institute of Applied Science and Technology Rangpur Bangladesh; ^4^ Department of Parasitology Bangladesh Agricultural University Mymensingh Bangladesh

**Keywords:** antimicrobial resistance (AMR), antimicrobial stewardship, Bangladesh, KAP (knowledge, attitude, and practices), livestock farming

## Abstract

Antimicrobial resistance (AMR) is a critical global health threat, intensified in low‐ and middle‐income countries by rampant antibiotic misuse in livestock and poultry. This study assessed the knowledge, attitudes, and practices (KAP) of 537 farmers in Rangpur, Bangladesh, to identify drivers and pathways of high‐risk antimicrobial use (AMU). Data on demographics, farm characteristics, and AMR‐related KAP scores were collected, with disease treatments mapped to WHO AWaRe classifications. Logistic regression identified predictors of responsible practices, while additional analyses and visualizations explored patterns of antimicrobial use and misuse pathways. Results showed that 42.9% of antibiotic use was high‐risk, with nearly half of diseases treated using Critically Important Antimicrobials (CIAs) from the Watch or Reserve groups, intended primarily for human medicine. Colistin and ciprofloxacin, last‐resort drugs for human health, were commonly used for routine poultry diseases, raising serious public health concerns. Three misuse pathways emerged: (i) antibiotics applied to viral diseases, (ii) reliance on Watch/Reserve antibiotics for bacterial infections, and (iii) antibiotic use for parasitic diseases. Paravets and veterinarians influenced 76.2% of prescribing decisions, underscoring their pivotal role. AMR training was associated with more responsible practices, yet high practice scores did not consistently align with knowledge or attitudes, revealing a gap between behavior and awareness. Immediate One Health stewardship interventions combining regulatory enforcement, improved diagnostics, and sweeping educational reform are essential to reduce AMR risks and safeguard public health in Bangladesh.

## Graphical abstract

Surveying 537 farmers in Bangladesh revealed widespread high‐risk antibiotic use, including misuse of critically important antimicrobials and treatment of non‐bacterial diseases. The findings highlight a major knowledge‐practice gap and emphasize the need for improved diagnostics, stronger regulation, and a One Health‐based antimicrobial stewardship approach.

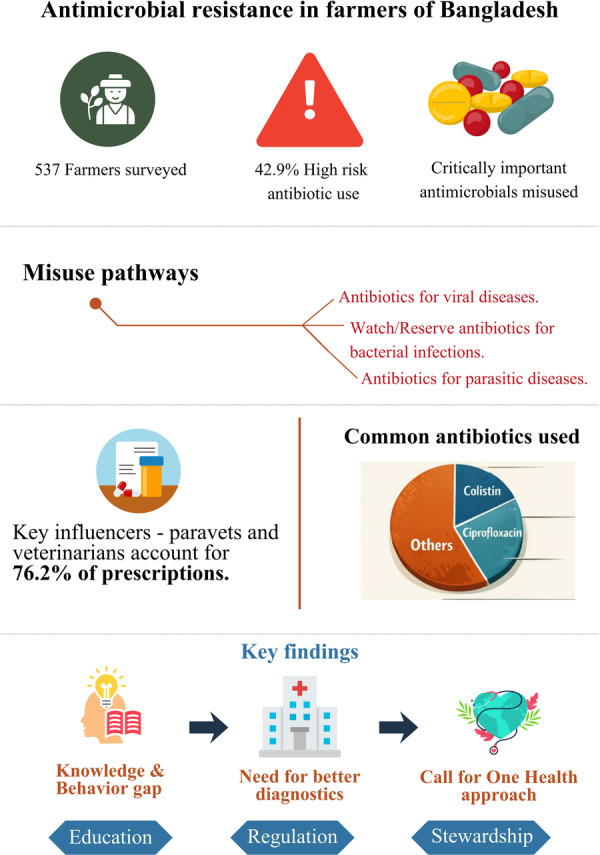

## 1 Introduction

Antimicrobial resistance (AMR) represents an unparalleled and urgent global health crisis. It threatens the efficacy of modern medicine and imposes an immediate, growing burden on human and animal health systems worldwide (WOAH 2024). The emergence and rapid spread of AMR are intrinsically linked to the unchecked overuse and misuse of antimicrobials across human, animal, and environmental sectors, underscoring the need for a rapid One Health response (WOAH 2024). Against this urgent backdrop, the livestock and poultry sectors are major contributors to the quickly escalating global AMR burden, largely due to widespread antibiotic use for disease prevention, treatment, and, in many regions, growth promotion (Van Boeckel et al. 2015).

The challenge of AMR is especially acute in low‐ and middle‐income countries (LMICs). It presents an urgent and escalating threat. High infectious disease prevalence, limited veterinary oversight, and easy access to over‐the‐counter antibiotics fuel widespread misuse (Tasmim et al. 2023). Bangladesh's rapidly expanding livestock and poultry sectors increase the stakes for food security and economic growth. Studies consistently report rampant and irrational use of antibiotics in commercial farming, often without veterinary prescription. This misuse is driven by poor biosecurity, pressure to produce rapidly, and a lack of farmer awareness (Masud et al. 2020; Imam et al. 2020). It accelerates the emergence of antibiotic‐resistant bacteria in poultry and dairy products. This creates a serious public health threat through the food chain (Al Amin et al. 2020).

To design effective interventions, it is essential to understand the behavioral and structural drivers of antimicrobial use. Knowledge, Attitude, and Practices (KAP) studies are fundamental for evaluating the understanding, perceptions, and behaviors of key stakeholders such as farmers regarding AMR (Islam et al. 2022). Moreover, responsible antimicrobial practices may be influenced by a combination of factors, including educational background, farm characteristics, economic constraints, and access to veterinary advisory services (Hassan et al. 2021).

Despite growing research on antimicrobial use in Bangladesh, region‐specific evidence on farmers' behavioral drivers remains limited, particularly in northern agricultural regions such as Rangpur. Rangpur district is an important livestock production area where both dairy and poultry farming play significant roles in local livelihoods and food supply. However, little empirical information is available regarding farmers' knowledge, attitudes, and practices related to antimicrobial use and resistance in this region. Understanding these behavioral and socio‐demographic determinants is important for designing targeted extension programs, veterinary advisory services, and antimicrobial stewardship interventions.

Therefore, the primary objective of this study was to assess behavioral drivers of antimicrobial use through a Knowledge‐Attitude‐Practice (KAP) framework of dairy and poultry farmers regarding AMR in Rangpur district, Bangladesh. Specifically, the study aimed to:

i. Assess the levels of knowledge, attitudes, and practices related to antimicrobial use and AMR among dairy and poultry farmers.
ii. Identify the sociodemographic and farm‐related factors (e.g., education, farm type, AMR training) associated with responsible antimicrobial practices.
iii. Examine the interrelationships between farmers' knowledge, attitudes, and practices to identify key behavioral gaps and potential intervention points for antimicrobial stewardship programs.

Unlike many previous antimicrobial use studies in Bangladesh that have focused primarily on single livestock sectors or descriptive antibiotic usage patterns, the present study provides a comparative assessment of dairy and poultry farmers' knowledge, attitudes, and practices regarding antimicrobial use and AMR within the same geographic region. By integrating composite KAP scoring, multivariable regression analysis, and behavioral pathway visualization, this study identifies key socio‐demographic and farm‐level determinants that influence responsible antimicrobial practices among farmers. The findings provide empirical evidence on behavioral drivers of antimicrobial misuse in a major agricultural region of northern Bangladesh, offering practical insights for policymakers, veterinary services, and extension programs aiming to strengthen antimicrobial stewardship within livestock production systems. By highlighting the gap between awareness and responsible practice, this study contributes to the growing body of evidence supporting behavior‐focused interventions within a One Health framework to mitigate AMR risks in food‐producing animal systems.

## 2 Materials and Methods

### 2.1 Study Area and Population

**Figure 1:**
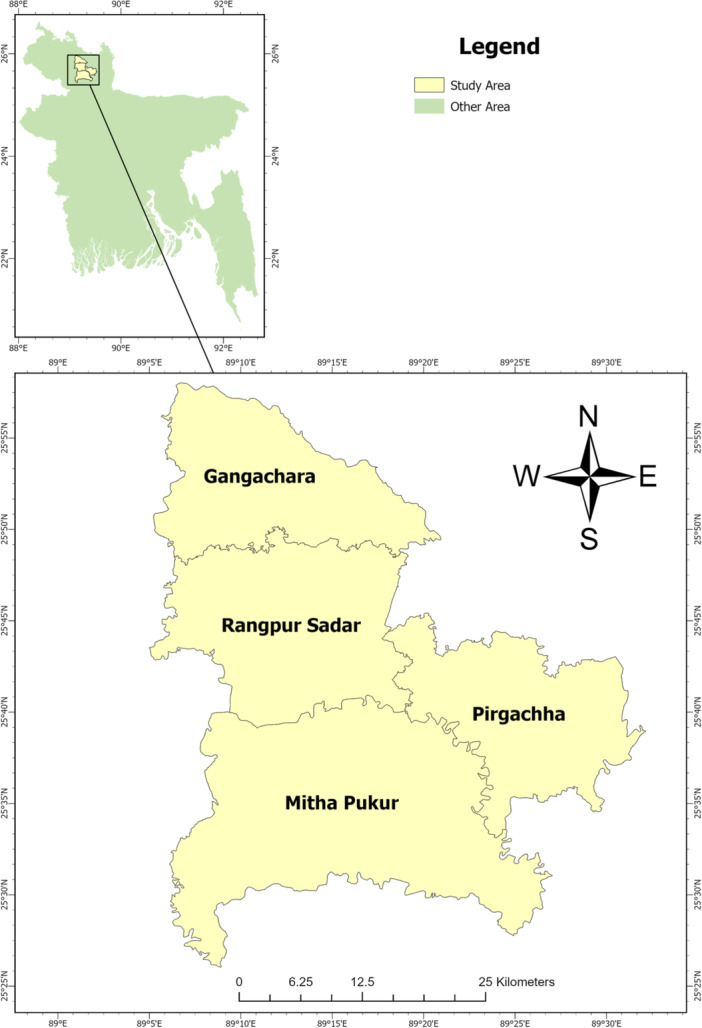
Map of the study area showing the four selected upazilas‐ Gangachara, Mithapukur, Rangpur Sadar, and Pirgacha in Rangpur District, Northern Bangladesh. The locations represent diverse agro‐ecological zones and farming systems included in the study.

The study was conducted between July 2023 and June 2025 in four upazilas of Rangpur district, Bangladesh: Gangachara, Mithapukur, Sadar, and Pirgacha (Figure 1). These sites were selected to represent diverse agro‐ecological zones and farming systems. Rangpur is a major hub for livestock and poultry production in northern Bangladesh. Smallholder and medium‐scale farms here contribute substantially to local food security and income.

The study population comprised livestock and poultry farmers actively engaged in farm management and decision‐making regarding animal health and antimicrobial use. Both dairy and poultry producers were included to capture variation in knowledge, attitudes, and practices (KAP) across farm types.

### 2.2 Sample Size Calculation

The required sample size was estimated using the formula for a single population proportion, a standard approach in cross‐sectional studies (Cochran, 1977; Daniel, 1999). Assuming an expected proportion of 50% for appropriate practices among farmers (to maximize sample size), a 95% confidence level, and a 5% margin of error, the minimum sample size was calculated to be approximately 385.

To account for potential non‐response and incomplete data, a 10% contingency was added, yielding a target of 422 farmers. Ultimately, 537 farmers were enrolled to enhance statistical power and ensure broad representation.

### 2.3 Study Design and Sampling

A cross‐sectional survey was conducted among livestock farmers in the selected upazilas. A total of 537 farmers were enrolled using stratified random sampling. This ensured balanced representation across farm types (dairy, poultry, mixed) and farm sizes. Eligibility criteria included active involvement in farm management and antimicrobial use decisions. Farmers unwilling to participate or unable to provide informed consent were excluded.

### 2.4 Data Collection

A structured questionnaire was developed based on validated KAP frameworks and adapted to the local context. The instrument included sections on:

- Socio‐demographic characteristics (age, education, farm type, farm size, AMR training exposure)
- Knowledge items on antimicrobial use and resistance
- Attitude items assessing perceptions and beliefs about AMR risks
- Practice items evaluating actual behaviors related to antimicrobial administration and farm biosecurity.

The questionnaire was pre‐tested with 30 farmers to ensure clarity and cultural appropriateness. Data were collected through face‐to‐face interviews by trained enumerators.

### 2.5 Statistical Analysis

Data were analyzed using SPSS and Python software. Descriptive statistics were used to summarize socio‐demographic characteristics and KAP distributions among farmers.

Chi‐square (*χ* ^2^) tests were performed to examine associations between categorical variables, including education level, farm type, farm size, age group with KAP outcomes. Variables that showed significant associations were further evaluated in multivariable logistic regression models.

Multivariable logistic regression was used to identify predictors of responsible antimicrobial practices. The dependent variable was high practice score, while independent variables included age, education level, farm type, farm size, and exposure to AMR training. Adjusted odds ratios (AORs) with 95% confidence intervals (CIs) were calculated. Selected visualization such as stacked bar charts, forest plots, Venn diagrams, and Sankey diagrams were used to illustrate the distribution of KAP categories, regression outcomes, overlaps among KAP components, and antimicrobial misuse pathways.

Data visualization techniques, including Venn diagrams, proxy correlation analysis, and Sankey diagrams, were applied to complement tabular results and facilitate interpretation of overlaps, correlations, and flow patterns among antimicrobial use practices and advisory sources.

## 3 Results

### 3.1 Socio‐Demographic Characteristics of Respondents

A total of approximately 537 livestock and poultry farmers participated in the study. The majority of respondents were male (89.0%, 95% CI: 86.1–91.4) and married (96.5%). Most households had four members (87.5%), and nearly all farmers had no formal education (93.9%). Household income was concentrated in the 200,000‐400,000 BDT (Bangladeshi Taka) range (99.1%). Farm sizes were predominantly medium (71.1%), with smaller proportions operating small (12.3%) or large farms (16.6%). Age distribution was skewed toward middle age (35–50 years), with significant variation across categories (*χ* ^2^ = 40.6, *p* = 0.004). Education, family size, and farm size also showed statistically significant associations with responsible antimicrobial use (*p* < 0.001) (Table 1).

**Table 1:** Socio‐demographic characteristics of livestock and poultry farmers (*n* = 537).

Variable	Category	*n*	% (95% CI)	*χ*² value	*p*‐value
Age group (years)	Young (< 35)	69	12.8 (10.2–15.9)	40.6	0.004
Age group (years)	Middle (35–50)	294	54.7 (50.4–59.0)	40.6	0.004
Age group (years)	Older (≥ 51)	174	32.4 (28.4–36.7)	40.6	0.004
Gender	Male	478	89.0 (86.1–91.4)	0.04	0.834
Gender	Female	59	11.0 (8.6–13.9)	0.04	0.834
Marital status	Married	518	96.5 (94.5–97.7)	0.00	1.000
Marital status	Unmarried	19	3.5 (2.3–5.5)	0.00	1.000
Family size	1–3 members	37	6.9 (5.0–9.4)	116.3	< 0.001
Family size	4 members	470	87.5 (84.5–90.1)	116.3	< 0.001
Family size	≥ 5 members	30	5.6 (3.9–8.0)	116.3	< 0.001
Education	No formal	504	93.9 (91.5–95.6)	106.3	< 0.001
Education	Primary	29	5.4 (3.8–7.6)	106.3	< 0.001
Education	Secondary	4	0.7 (0.3–1.9)	106.3	< 0.001
Household income	< 200,000 BDT	3	0.6 (0.2–1.6)	0.06	0.972
Household income	200–400,000 BDT	532	99.1 (97.8–99.6)	0.06	0.972
Household income	> 400,000 BDT	2	0.4 (0.1–1.3)	0.06	0.972
Farm size	Small (< 10)	66	12.3 (9.7–15.5)	129.5	< 0.001
Farm size	Medium (10–100)	382	71.1 (67.2–74.8)	129.5	< 0.001
Farm size	Large (> 100)	89	16.6 (13.6–20.1)	129.5	< 0.001

### 3.2 Knowledge, Attitudes, and Practices (KAP) Related to Antimicrobial

Overall, farmers demonstrated high awareness of AMR risks (e.g., misuse leads to AMR, 84.4% agreement) but low adherence to responsible practices such as consulting veterinarians (10.8%), keeping records (10.8%), or observing withdrawal periods (11%). Preventive measures like vaccination (13.2%) and isolation of sick animals (6.7%) were rarely practiced. Conversely, nearly all respondents agreed that drug sellers often recommend antibiotics unnecessarily (100%) and that family pressure for quick treatment is universal (100%). Training and demonstration farms (92.6%) and premium pricing for antibiotic‐free products (88.6%) were widely perceived as strong motivators for change (Table 2).

**Table 2:** Knowledge, attitudes, and practices (KAP) related to antimicrobial use among livestock and poultry farmers (*n* = 537).

Item code	Statement (Short form)	Mean (SD)	% Agree (4 or 5)
B_S1	Antibiotics treat bacterial infections	3.74 (0.81)	83.8%
B_S2	Antibiotics can be used for growth promotion	2.97 (0.66)	11.2%
B_S3	Misuse of antibiotics can lead to AMR	4.52 (1.12)	84.4%
B_S4	AMR affects both animals and humans	3.09 (0.71)	13.8%
B_S5	Withdrawal periods are necessary	2.37 (0.91)	13.8%
B_S6	Over‐the‐counter antibiotics are commonly used	3.06 (0.76)	13.0%
B_S7	Consult a veterinarian before using antibiotics	2.99 (0.65)	10.8%
B_S8	Awareness of government guidelines	2.26 (0.78)	10.6%
C_S1	Antibiotics only when prescribed by vet	2.30 (0.84)	11.5%
C_S2	Reducing antibiotic use improves long‐term health	3.71 (0.84)	83.6%
C_S3	AMR is a serious public health threat	2.39 (0.93)	13.8%
C_S4	Farmers should receive training	2.28 (0.89)	14.0%
C_S5	Willing to adopt alternatives	2.32 (0.85)	13.0%
D_P1	Use antibiotics without vet consultation	2.39 (0.90)	15.3%
D_P2	Keep records of antibiotic use	2.95 (0.74)	10.8%
D_P3	Observe withdrawal periods	1.49 (1.17)	11.0%
D_P4	Use herbal/traditional remedies	1.59 (1.28)	13.0%
D_P5	Vaccinate livestock regularly	1.60 (1.27)	13.2%
D_P6	Isolate sick animals	1.41 (0.97)	6.7%
D_P7	Clean/disinfect housing regularly	4.56 (1.06)	88.6%
D_P8	Participate in AMR awareness programs	4.43 (1.21)	83.2%
E_S1	Confident to manage diseases without routine antibiotics	1.45 (1.05)	8.4%
E_S2	Knowledge to choose appropriate antibiotics/doses	3.01 (0.77)	11.7%
E_S3	Can afford veterinary services	1.58 (1.23)	13.4%
E_S4	Access to alternatives	2.26 (0.92)	9.7%
E_S5	Can follow withdrawal periods	2.26 (0.95)	11.0%
F_S1	Community follows vet prescriptions	1.60 (1.24)	14.0%
F_S2	Family expects quick treatment	4.85 (0.35)	100%
F_S3	Advisors recommend prudent use	3.06 (0.82)	12.8%
F_S4	Drug sellers recommend antibiotics unnecessarily	4.88 (0.33)	100%
G_B1	Lack of affordable vet service prevents prudent use	2.97 (0.73)	10.6%
G_B2	Lack of AMR knowledge prevents better practices	1.46 (1.00)	8.9%
G_B3	Market pressure disincentivizes withdrawal	3.03 (0.78)	13.4%
G_B4	Cheap antibiotics encourage misuse	2.24 (0.77)	11.4%
G_I5	Subsidized vet services would help	3.03 (0.62)	12.1%
G_I6	Training/demo farms motivate adoption	4.70 (0.78)	92.6%
G_I7	Premium price for antibiotic‐free products encourages	3.91 (0.56)	88.6%
G_C1–C12	Constraints (cost, access, infrastructure, training)	4.7–4.8 (0.7)	88–90%

### 3.3 Associations Between Sociodemographic Factors and KAP Outcomes

Cross‐tabulation and chi‐square analyses were conducted to examine associations between sociodemographic variables and KAP outcomes (Table 3). Education level showed a significant association with knowledge scores (*χ* ^2^ = 18.7, *p* < 0.001), with farmers having secondary education demonstrating higher knowledge levels compared with those without formal education.

**Table 3:** Association between composite KAP categories and socio‐demographic factors (*n* = 537).

Factor	Category	Knowledge (Low/Moderate/High)	Attitude (Low/Moderate/ High)	Practice (Low/Moderate/ High)	*χ*² value	*p*‐value
Education	No formal education	23.5/53.8/22.7	11.4/88.6/0.0	0.0/36.9/63.1	18.7	*p* < 0.001
Education	Primary	Higher knowledge & attitudes	Slightly better practices		18.7	*p* < 0.001
Education	Secondary	Highest knowledge (≈ 4.1 mean)	Moderate attitudes	Lower practices	18.7	*p* < 0.001
Training	No AMR training	2.92 mean (mostly Moderate)	2.38 mean (Moderate)	3.98 mean (High)	12.4	*p* < 0.001
Farm type	Dairy	Lower knowledge (2.75 mean)	Lower attitudes (2.28 mean)	Highest practices (4.04 mean)	26.1	*p* < 0.001
Farm type	Mixed	Higher knowledge (3.73 mean)	Better attitudes (2.98 mean)	Lower practices (3.69 mean)	26.1	*p* < 0.001
Farm type	Poultry	Highest knowledge (4.39 mean)	Highest attitudes (3.11 mean)	Lowest practices (3.50 mean)	26.1	*p* < 0.001

Training exposure was also significantly associated with improved knowledge and attitudes toward AMR (*χ* ^2^ = 12.4, *p* < 0.001). Farm type exhibited strong differences across KAP components (*χ* ^2^ = 26.1, *p* < 0.001). Poultry farmers demonstrated higher knowledge and attitude levels, while dairy farmers showed comparatively stronger practice scores.

In contrast, age did not show a statistically significant association with knowledge (*p* = 0.064) or practice (*p* = 0.239) outcomes, suggesting that structural and educational factors may play a more prominent role in shaping antimicrobial use behaviors among farmers.

### 3.4 Regression Analysis

Multivariable logistic regression analysis identified several factors significantly associated with responsible antimicrobial practices among farmers (Table 4).

**Table 4:** Logistic regression of predictors of high practice scores (*n* = 537).

Predictor	Adjusted odds ratio (AOR)	95% CI	*p*‐value
Age (continuous)	1.02	0.99–1.05	0.12
Education			
Primary versus No formal	1.45	1.10–1.92	0.008
Secondary versus No formal	2.30	1.15–4.61	0.018
Farm type			
Poultry versus Dairy	1.85	1.20–2.85	0.005
Mixed versus Dairy	1.40	0.95–2.06	0.09
Farm size (continuous)	1.01	1.00–1.02	0.04
AMR training (Yes vs. No)	2.10	1.45–3.05	

Farmers with secondary education were more than twice as likely to demonstrate high practice scores compared with those without formal education (AOR = 2.30, 95% CI: 1.15–4.61). Poultry farmers also showed significantly higher odds of reporting responsible practice compared with dairy farmers (AOR = 1.85, 95% CI: 1.20–2.85).

Farm size showed a modest but significant association with practice outcomes (AOR = 1.01 per unit increase, *p* = 0.04). Importantly, farmers who had received AMR training were approximately twice as likely to report high practices (AOR = 2.10, 95% CI: 1.45–3.05, *p* < 0.001).

Age was not significantly associated with antimicrobial practices, suggesting that educational exposure and farm characteristics are more influential determinants of antimicrobial stewardship behavior.

### 3.5 Overlap Between Knowledge, Attitude, and Practice

**Figure 2:**
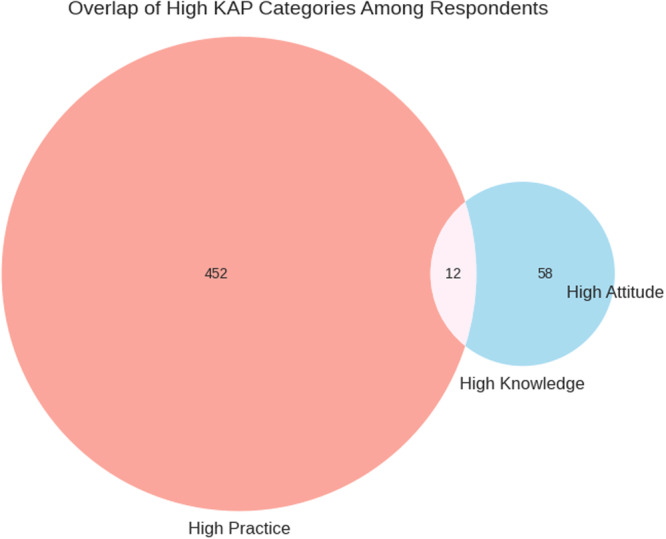
A Venn diagram showing the knowledge, attitude, and practice level among the participants.

The Venn diagram illustrates the overlap between farmers' knowledge, attitudes, and practices regarding AMR (Figure 2). While a considerable proportion of farmers exhibited high practice scores, fewer respondents simultaneously demonstrated high levels of knowledge and positive attitudes.

Only a small subset of farmers achieved high scores across all three KAP dimensions, highlighting a gap between awareness and behavioral implementation. This finding suggests that many practices may be driven by routine husbandry norms or external influences rather than informed decision‐making.

### 3.6 Source of Antimicrobial Advice, Purchase and High‐Risk Antibiotic Use

Farmers reported multiple sources of advice and access to antimicrobials (Table 5). Paravets represented the most common advisory source (39.9%), followed by veterinarians (36.3%). Drug sellers (15.4%) and private practitioners (7.3%) also contributed to antimicrobial guidance, while other sources accounted for a small proportion (1.2%). These findings indicate that antimicrobial access and treatment advice are obtained from both formal veterinary services and informal providers within the livestock production system.

**Table 5:** Source of antimicrobial advice and purchase in study areas.

Source category	Avg. weekly patients	Percentage (%)
Paravet	407	39.90
Veterinarian	370	36.27
Drug seller	157	15.39
Private practitioner	74	7.25
Other	12	1.18

**Figure 3:**
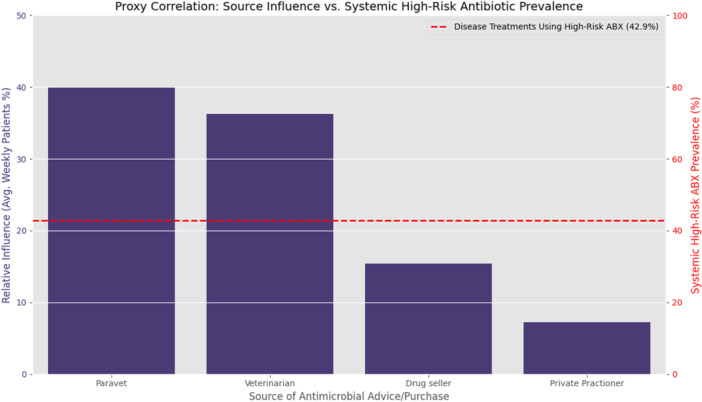
The visualization provides a proxy for the correlation between the influence of different advice sources and the systemic prevalence of high‐risk antibiotic use.

The prevalence of systemic high‐risk antibiotic use was 42.9%, indicating that nearly half of common livestock and poultry diseases are treated with “Watch” or “Reserve” antimicrobials. The visualization further shows that paravets (39.9%) and veterinarians (36.3%) together account for 76.2% of weekly patient interactions. These dominant advisory sources operate in a high‐risk prescribing environment where critically important antimicrobials (CIAs) are frequently used, underscoring the need for targeted stewardship interventions that focus on paravets and veterinarians to ensure informed and responsible prescribing practices (Figure 3).

### 3.7 Classification of Antibiotics Used in the Study Area

Antibiotics reported by farmers were classified according to WHO AWaRe and WOAH guidelines, which categorize antimicrobials based on their importance in human medicine (Table 6). Several commonly used antibiotics belonged to the Watch category, including fluoroquinolones (ciprofloxacin, enrofloxacin) and macrolides (tylosin, erythromycin). Phenicols such as florfenicol were also reported. A small proportion of respondents indicated the use of colistin, which is classified in the Reserve group and is restricted in many countries due to its importance as a last‐line treatment in human medicine.

**Table 6:** High‐risk antibiotics identified in the study area.

Antibiotic class	Specific antibiotics	Classification	Notes on misuse and AMR risk
Fluoroquinolones	Ciprofloxacin, Enrofloxacin	Watch	Widespread use in poultry; resistance frequently reported.
Polymyxins	Colistin (Colistin Sulfate)	Reserve	Banned but still used; highest risk for AMR development.
β‐lactams (Cephalosporins)	Ceftriaxone (3rd Gen)	Watch	Used as last‐resort; inappropriate for routine animal use.
Macrolides	Tylosin, Erythromycin	Watch	Risk of cross‐resistance; used for Mycoplasma infections.
Aminoglycosides	Gentamicin	Watch	Used for severe infections; high resistance potential.
Phenicols	Florfenicol	Watch	Broad‐spectrum use; contributes to resistance selection.

**Figure 4:**
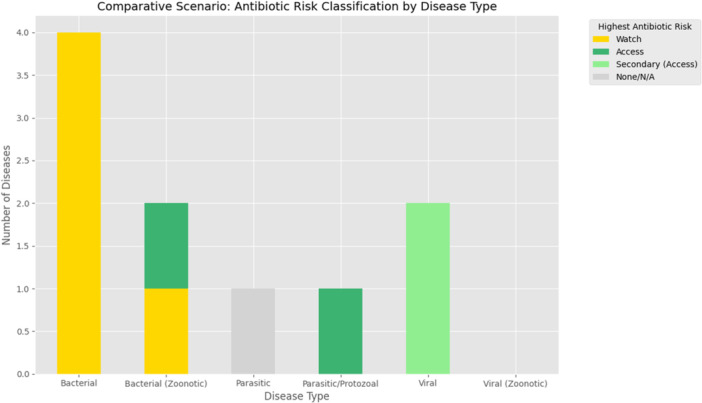
Comparative distribution of antibiotic risk classifications across different disease types used in the study area.

The comparative distribution of antibiotic risk classifications across different disease types revealed that bacterial diseases were predominantly treated with Watch‐category antibiotics, representing the highest‐risk antimicrobial use. In contrast, zoonotic bacterial diseases showed a mix of Watch and Access antibiotics. Parasitic and protozoal diseases were mainly associated with Access‐category antibiotics, while viral diseases were largely linked to secondary (Access) treatments, indicating limited direct antibiotic use (Figure 4).

### 3.8 Mapping of Diseases to Antibiotic Treatments

Table 7 presents the relationship between commonly reported livestock diseases and the antibiotics used for treatment. Bacterial diseases such as colibacillosis and salmonellosis were frequently treated with Watch‐group antibiotics. In addition, antibiotics were sometimes used in the management of viral diseases, including Newcastle disease and infectious bursal disease, typically for the prevention or treatment of secondary bacterial infections.

**Table 7:** Diseases treated with high‐risk antibiotics.

Disease name	Type	Affected species	Common antibiotic treatment	Highest risk classification
Bacterial Diarrhea	Bacterial	Goats, Sheep, Poultry	Amoxicillin, Tetracyclines, Florfenicol	Watch
Pneumonia	Bacterial	Goats, Sheep, Poultry	Tetracyclines, Amoxicillin, Enrofloxacin	Watch
Salmonellosis	Bacterial (Zoonotic)	Poultry, Humans	Florfenicol, Neomycin, Ciprofloxacin, Colistin	Watch
Colibacillosis	Bacterial	Poultry	Ciprofloxacin, Colistin, Gentamicin, Doxycycline	Watch
Mycoplasmosis	Bacterial	Poultry	Tylosin, Erythromycin, Tiamulin, Tetracyclines	Watch

### 3.9 Misuse Pathway Analysis

**Figure 5:**
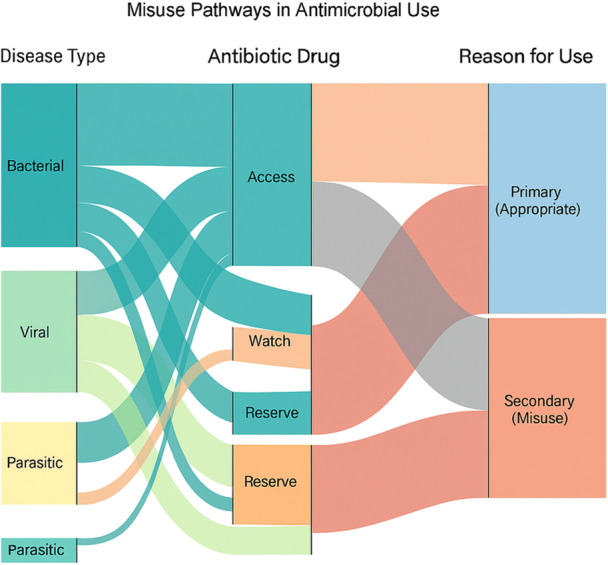
Sankey diagram (A, B) of misuse pathways in antimicrobial practices. This diagram illustrates the flow of antimicrobial use from disease type (left column) through antibiotic risk classification (middle column: Access, Watch, Reserve) to the reason for use (right column: Primary Treatment or Secondary Use/Misuse). The width of each band represents the number of disease instances associated with that pathway.

The Sankey diagram (Figure 5) illustrates the flow from disease categories to antibiotic risk classifications and final use patterns. Three major pathways were observed:

1. Bacterial disease treatment, where Watch‐group antibiotics were commonly used.
2. Antibiotic use in viral diseases, where drugs were applied as secondary treatments.
3. Antimicrobial use associated with parasitic infections, including treatment of tick‐borne diseases using tetracyclines.

These patterns highlight several areas where antimicrobial use practices could potentially be optimized through improved diagnostic support and antimicrobial stewardship interventions.

## 4 Discussion

This study provides important insights into farmers' knowledge, attitudes, and practices (KAP) regarding antimicrobial use in livestock production systems in Bangladesh. The findings highlight several structural and behavioral factors influencing antimicrobial use, including education level, farm type, access to veterinary services, and participation in AMR training programs. Although many farmers demonstrated moderate awareness of AMR, responsible antimicrobial practices were not consistently followed, indicating a knowledge–practice gap widely reported in Bangladesh and other LMICs (Hassan et al. 2021; Islam et al. 2022; Tasmim et al. 2023; Chowdhury et al. 2022).

Socio‐demographic characteristics reflected typical rural livestock production patterns, with male household members primarily managing livestock as an important income source (Islam et al. 2022; Tasmim et al. 2023; Elias 2024; & Sarker 2024). Limited formal education may hinder adoption of evidence‐based practices, whereas higher education levels were associated with more responsible AMU, emphasizing the role of targeted awareness programs (Imam et al. 2020; Hassan et al. 2021).

Farm characteristics also influenced AMU. Poultry and more commercialized farms exhibited higher antimicrobial use due to intensive production practices, increased disease risk, and economic pressures to maintain productivity. Generational differences further shaped behaviors, with younger farmers more receptive to updated recommendations and older farmers relying on traditional practices (Islam et al. 2022; Chowdhury et al. 2022). Participation in AMR training programs strongly correlated with prudent antimicrobial practices, highlighting the importance of farmer education and extension initiatives for stewardship.

Antimicrobial use patterns in Rangpur reveal critical governance and behavioral drivers. The prevalence of systemic high‐risk antibiotics (42.9%) indicates that nearly half of common diseases are treated with Watch or Reserve antibiotics (critically important antimicrobials, CIAs), reflecting unsafe prescribing practices (Chowdhury et al. 2021). Paravets (39.9%) and veterinarians (36.3%) together account for over three‐quarters of weekly patient interactions, making them the primary sources of antimicrobial advice (Kalam et al. 2021; Matin et al. 2020; Azim et al. 2023). While veterinarians are legally authorized to prescribe antimicrobials, high‐risk prescribing persists due to diagnostic limitations, farmer pressure, and reliance on broad‐spectrum drugs (Orubu et al. 2021; Shano et al. 2024). Paravets and drug sellers frequently provide advice outside formal oversight and may promote broad‐spectrum antibiotics due to accessibility and commercial incentives (Masud et al. 2020; Tasmim et al. 2023). Strengthening diagnostic capacity, guideline adherence, regulatory enforcement, and stewardship training for both groups is essential to reduce CIA misuse (World Health Organization 2023).

Socio‐economic pressures further drive AMU, as farmers prioritize rapid disease treatment to minimize production losses. CIAs, including fluoroquinolones and, occasionally, colistin, are used despite regulatory restrictions, and antibiotics are often administered for viral diseases, providing no therapeutic benefit (Chowdhury et al. 2021; Islam et al. 2023). These findings underscore the need for One Health–oriented stewardship strategies, combining farmer training, accessible veterinary services, and stronger regulatory oversight.

Although this study did not include microbiological testing, recent evidence from Bangladesh supports the link between livestock AMU and AMR. Metagenomic studies of poultry farms and environmental surveillance have identified diverse Antibiotic resistance genes, including those conferring resistance to critically important antibiotics such as fluoroquinolones, aminoglycosides, and β‐lactams (Yasmin et al. 2025; Faruk et al. 2025; Islam et al. 2025; Rahman et al. 2025). These findings highlight livestock production systems as potential reservoirs of AMR. In addition, this study relied on self‐reported antimicrobial usage rather than pharmaceutical sales or prescription data; therefore, supply‐chain information and historical sales trends could not be independently verified. Future research integrating behavioral surveys with microbiological surveillance and genomic characterization of pathogens would provide stronger evidence linking antimicrobial use practices to local AMR dynamics within a One Health framework.

Overall, improving responsible AMU in livestock farming requires a multifaceted approach, integrating farmer education, strengthened veterinary services, targeted AMR training, and effective regulatory frameworks to support sustainable livestock production in Bangladesh.

## 5 Conclusion

This study provides critical insights into the sociodemographic determinants of antimicrobial use and AMR awareness among livestock and poultry farmers in northern Bangladesh. Farmers showed moderate knowledge and attitudes but generally high practices, reflecting a paradox between awareness and behavior. Education, farm type, and farm size were strongly associated with responsible practices, while age was not significant, underscoring the importance of structural rather than generational factors. Poultry farmers demonstrated higher knowledge and attitudes, whereas dairy farmers showed stronger practice adherence, indicating farm‐specific differences in stewardship.

Targeted interventions are needed to strengthen knowledge and attitudes through education, structured training, and regulation of informal drug markets, while leveraging existing positive practices. Integrating One Health approaches combining farmer training, veterinary oversight, and market incentives for antibiotic‐free products will be essential to align awareness with responsible antimicrobial use and reduce AMR risks in Bangladesh's livestock sector.

## Author Contributions

**Anju Man Ara:** conceptualization, methodology, investigation, formal analysis, writing – original draft (Lead); **Farjana Boby:** Investigation, data curation, writing – original draft (supporting). **Mohsin Taluckder:** conceptualization (supporting); supervision, writing – review and editing (supporting). **Md. Shahiduzzaman:** investigation, formal analysis, visualization, supervision, writing – review and editing (Lead).

## Funding

The authors have nothing to report.

## Ethics Statement

Ethical approval (No. IIAST/REC/2025‐07) was obtained from the IIAST Research Ethics Committee (REC). Informed consent was obtained from all animal owners and farm authorities prior to data collection. The objectives of the study, data collection procedures, and data confidentiality were explained in the local language (Bangla).

## Conflicts of Interest

The authors declare no conflicts of interest.

## Data Availability

The data that support the findings of this study are available on request from the corresponding author. The data are not publicly available due to privacy or ethical restrictions. Data will be available upon request to the corresponding author.
